# Supplementary figures and images for: Evolutionary dynamics of Respiratory Syncytial Virus in pre-pandemic, pandemic, and post-pandemic periods in Houston, Texas, USA
Source: bioRxiv. 2026 Jun 17:2025.08.06.668939. Preprint. [Version 2] doi: 10.1101/2025.08.06.668939 (PMC13308141; doi:10.1101/2025.08.06.668939)

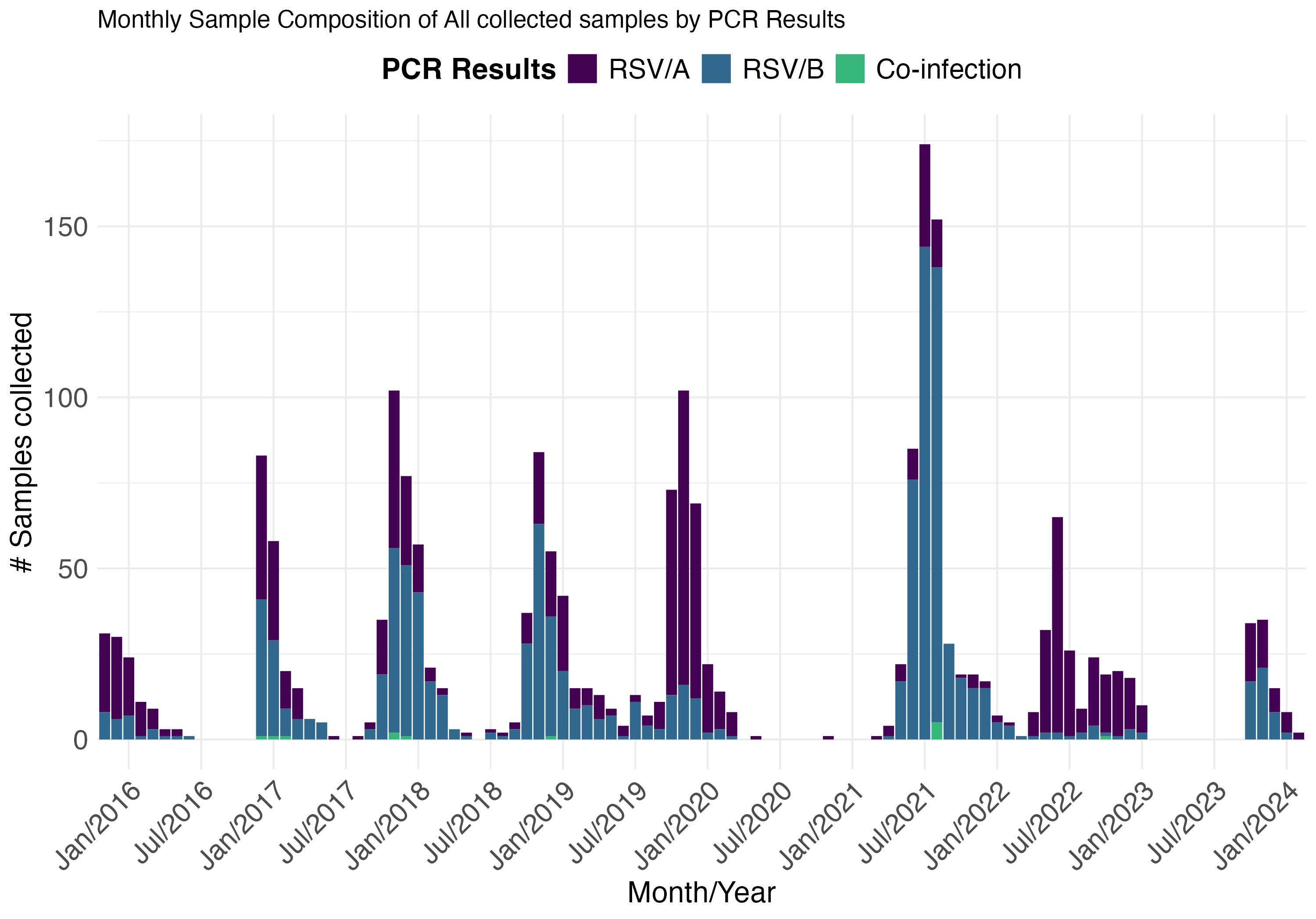

Supplement: Supplement 1 [file media-1.jpg]

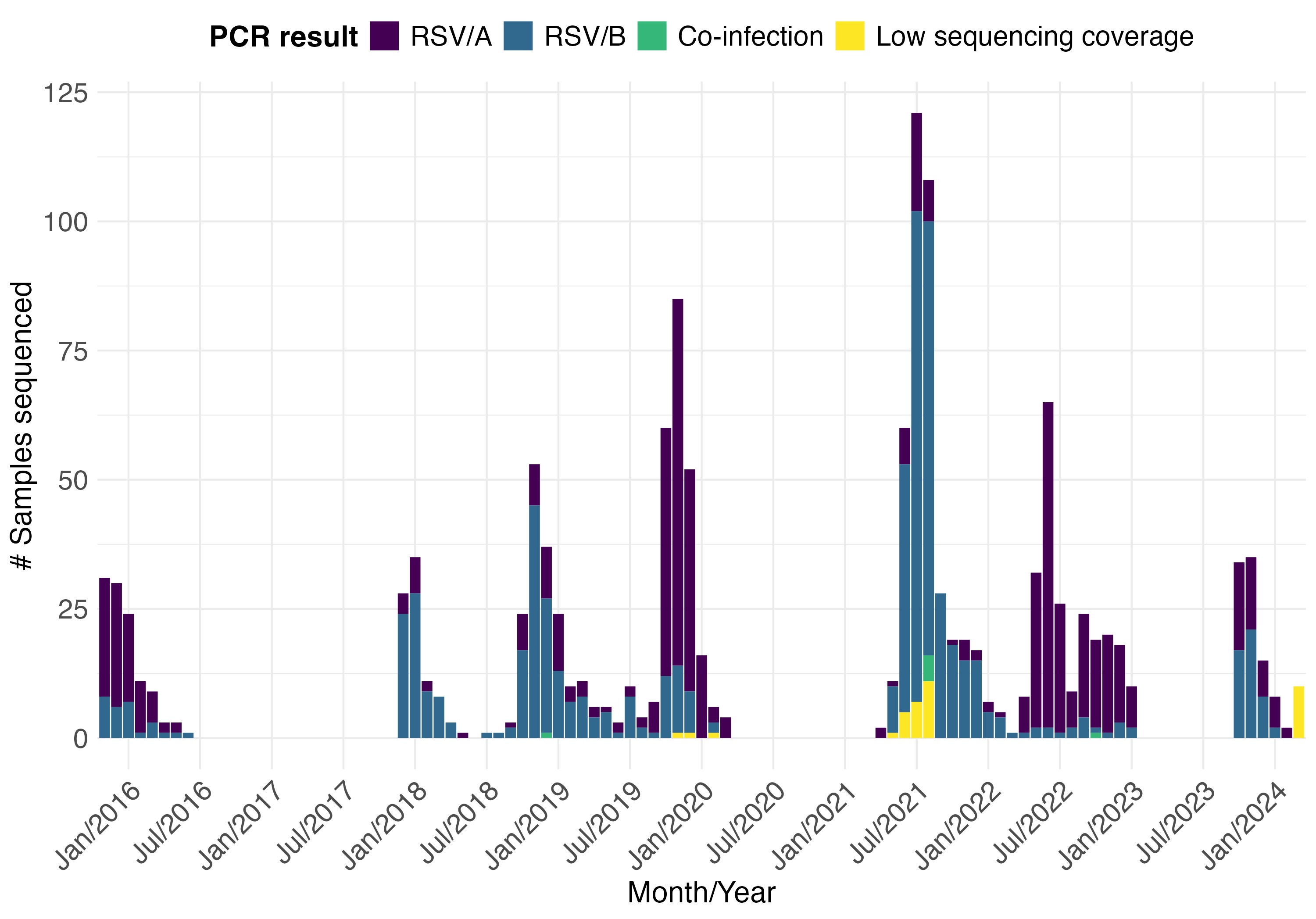

Supplement: Supplement 2 [file media-2.jpg]

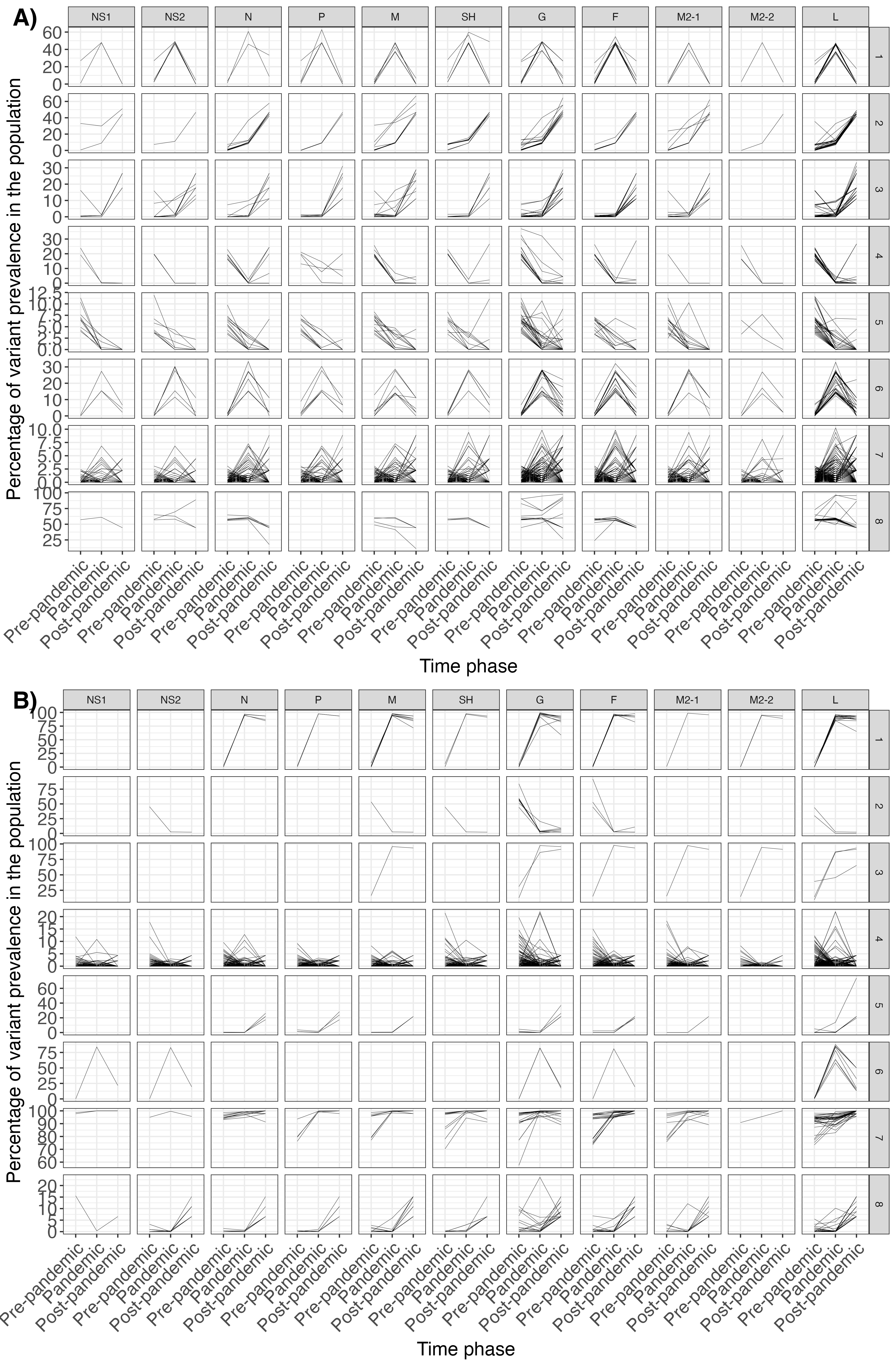

Supplement: Supplement 3 [file media-3.jpg]

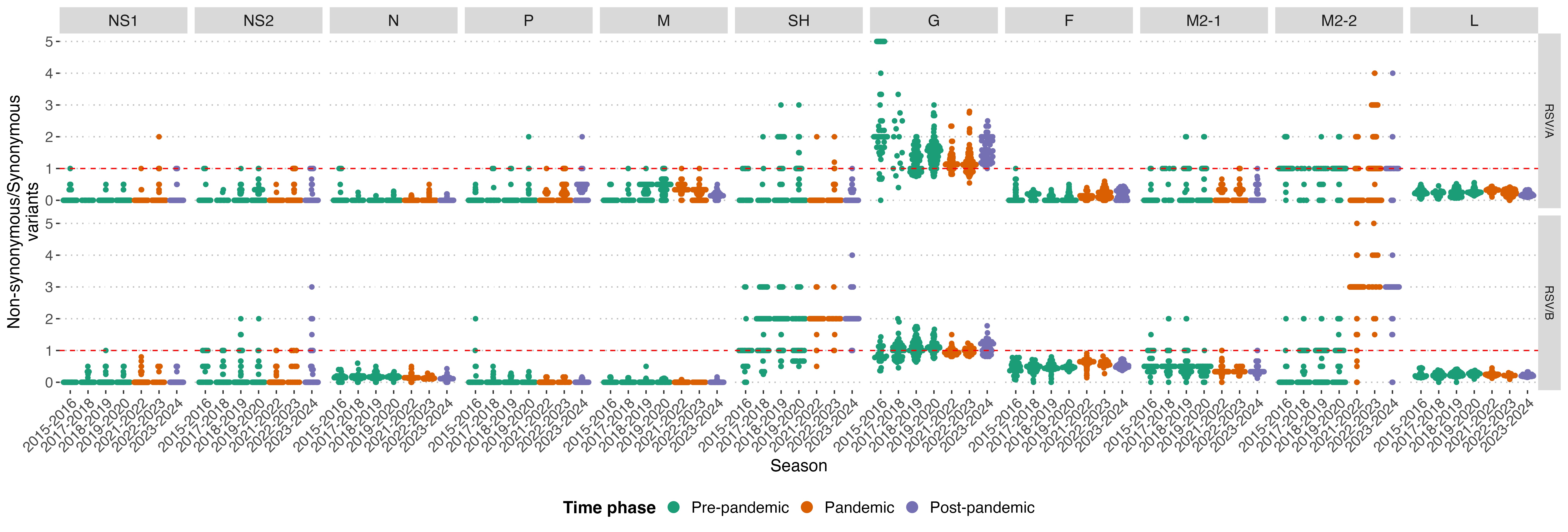

Supplement: Supplement 4 [file media-4.jpg]

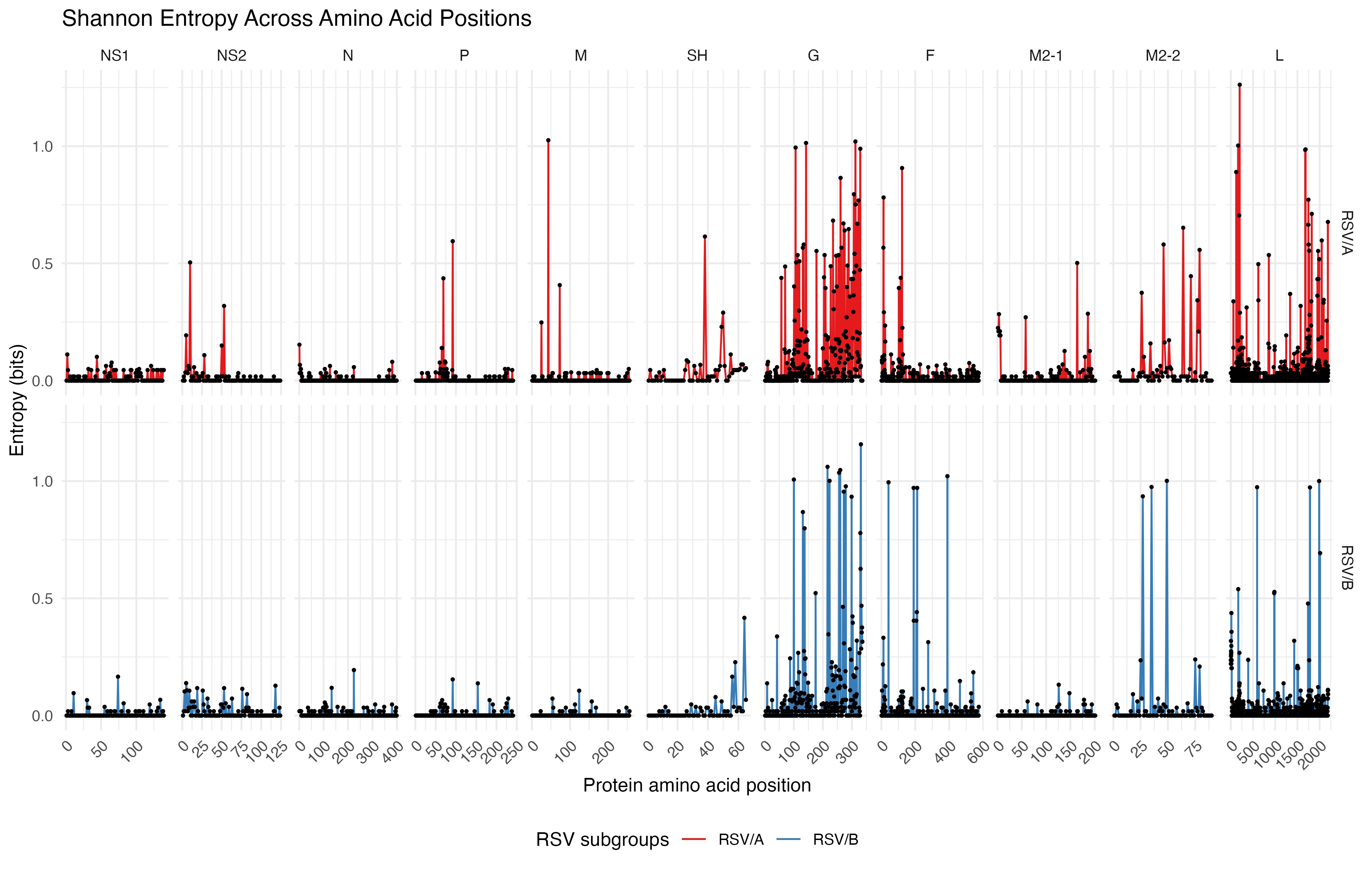

Supplement: Supplement 5 [file media-5.jpg]
